# Long-Term Clinical Outcomes After Percutaneous Coronary Intervention With Drug-Coated Balloon-Only Strategy in *de novo* Lesions of Large Coronary Arteries

**DOI:** 10.3389/fcvm.2022.882303

**Published:** 2022-07-14

**Authors:** Feng-Wang Hu, Shang Chang, Qian Li, Yong-Xiang Zhu, Xin-Yu Wang, You-Wei Cheng, Qi-Hua Zhou, Bing Liu, Javaid Iqbal, Xiao-Xia Tang, Yao-Jun Zhang

**Affiliations:** ^1^Department of Cardiology, Xuzhou Third People’s Hospital, Xuzhou Medical University, Xuzhou, China; ^2^Department of Cardiology, Xuzhou Renci Hospital, Xuzhou, China; ^3^Department of Cardiology, Peixian County Guotai Hospital, Xuzhou, China; ^4^Department of Cardiology, Sheffield Teaching Hospitals NHS Foundation Trust, Sheffield, United Kingdom

**Keywords:** drug-coated balloon, percutaneous coronary intervention, coronary bifurcation lesions, coronary *de novo* lesion, large coronary vessels

## Abstract

**Background and Objectives:**

Use of drug-coated balloon (DCB)-only strategy for revascularization of native large coronary artery lesions is on the rise. The long-term efficacy of this approach for bifurcation and non-bifurcation lesions remains unknown. We aim to assess the long-term clinical outcomes of DCB-only strategy for the treatment of *de novo* bifurcation and non-bifurcation lesions in large coronary arteries.

**Methods:**

This multicenter, prospective, observational study enrolled 119 patients with *de novo* coronary lesions in vessels ≥2.75 mm. The primary end point was the rate of clinically driven target lesion failure (TLF), a composite of cardiac death, target vessel myocardial infarction, and clinically driven target lesion revascularization (TLR). Patients were followed up for a median of 2 years.

**Results:**

Of 119 patients with 138 lesions, 66 patients (75 lesions) had bifurcation and 53 patients (63 lesions) had non-bifurcation lesions. Average reference vessel diameter was 3.1 ± 0.3 mm, and there was no difference in bifurcation and non-bifurcation group (3.0 ± 0.3 vs. 3.1 ± 0.3mm; *p* = 0.27). At 2-year follow-up, the TLF occurred in five (4.2%), TLR in four (3.4%), and target vessel revascularization (TVR) in five (4.2%) cases. The frequency of TLR and TVR was higher in the non-bifurcation group (*p* = 0.04 and 0.02, respectively), but there were no differences in TLF between the two groups (*p* = 0.17). The cumulative incidence of TLF (Kaplan–Meier estimates) was also not different in the two groups (log-rank *p* = 0.11).

**Conclusion:**

DCB-only strategy for *de novo* lesions in large coronary arteries appears to be safe and effective for both bifurcation and non-bifurcation lesions. Further randomized clinical trials are warranted to confirm the value of DCB-only strategy in *de novo* bifurcation lesions of large vessels.

## Introduction

Percutaneous coronary intervention (PCI) was historically performed without stent deployment, a technique that is currently referred to as plain old balloon angioplasty (POBA) (1). Outcomes of POBA were, however, compromised by acute vessel closure immediately and restenosis at follow-up (2). Coronary stents, initially bare metal and subsequently drug-eluting stents (DES), were, therefore, developed to overcome these issues. The vast majority of PCI procedures performed currently use DES. However, this leads to permanent metal implantation in the vessel with potential long-term consequences, including late stent thrombosis and restenosis. PCI with drug-coated balloons (DCBs) offers the advantage of leaving no metallic struts in the vessel and avoiding restenosis due to antiproliferative medication delivered by these devices (3, 4).

The DCB-only strategy has become an established treatment for in-stent restenosis (ISR) (4). A large number of clinical studies have also shown that DCB-only strategy provides optimal results in the treatment of native small-vessel disease (5). A long-term 5-year follow-up study has reported that DCB PCI for stable, *de novo*, coronary artery disease has similar results compared with second-generation non-paclitaxel stents (6). Recently, several observational studies have suggested that DCB-only PCI of *de novo* lesions in large coronary vessels is also safe and effective (7, 8). However, follow-up period in these studies is typically less than 12 months and proportion of coronary bifurcation lesions is small or undefined. Data for long-term efficacy of DCB-only strategy for native bifurcation and non-bifurcation lesions of large coronary vessels are currently limited. We, therefore, conducted this cohort study to determine long-term clinical outcomes of DCB-only therapy for *de novo* lesions of large coronary vessels. At the same time, we compared DCB-only PCI in bifurcation vs. non-bifurcation lesions.

## Materials and Methods

### Study Population

Patients were prospectively enrolled in three hospitals in the city of Huaihai, China (Xuzhou Third People’s Hospital, Pei County Guotai Hospital, Xuzhou Renci Hospital) from September 2017 to January 2020. Eligible patients were those with *de novo* lesions in large coronary vessels (reference vessel diameter ≥2.75 mm by visual estimation) and treated with DCB-only therapy. Bifurcation lesions were included if side branch vessel diameter ≥2.0 mm (target lesions in main branch) or ≥2.75 mm (target lesions in side branch). The major exclusion criteria included patients with cardiogenic shock or severe heart failure (New York Heart Association ≥III), estimated glomerular filtration rate <30 ml/min, ISR lesions, lesions located in left main coronary artery, and life expectancy <1 year. The study was approved by the local ethical review board and is registered with ClinicalTrials.gov (NCT04641468).

### Study Devices and Procedure

Paclitaxel-coated balloons were used, including Sequent^®^ Please (Braun, Germany) and Swide^®^ DCB (Shenqi Medical, China). DCB therapy was performed after optimal lesion preparation in compliance with the recommendations of the German Consensus Group on how to use DCBs in coronary artery disease (9). Standard semi-compliant balloons were used to dilate the target lesion. In the case of expansion failure of a semi-compliant balloon, a high-pressure non-compliant balloon or cutting and scoring balloon was used. If the final outcome of pre-dilation was acceptable (i.e., diameter stenosis <30% [by visual estimation] and thrombolysis in myocardial infarction [TIMI] flow grade 3), the DCB was used with length exceeding each edge of the lesion by at least 2 mm and inflated by nominal pressure for at least 30 s. The DCB diameters were sized to the reference vessel diameters with a balloon-to-vessel ratio of 0.8–1.0. Bifurcations could be treated with DCB angioplasty in the main and side branch and with kissing DCB if needed. Bailout stent implantation was considered in case of coronary dissection greater than or equal to type C, leading to vessel closure or residual stenosis of the treated lesion >30% after balloon angioplasty.

All patients, except one in the non-bifurcation group with aspirin-induced gastric problems, received dual antiplatelet therapy (DAPT) with aspirin (100 mg/day) and clopidogrel (75 mg daily) or ticagrelor (90 mg two times per day). Loading with aspirin (300 mg), clopidogrel (300 mg), or ticagrelor (180 mg) was administered if necessary. Intravenous heparin was used for procedural anticoagulation to maintain an activated clotting time between 250 and 300 s. In the postoperative period, DAPT was recommended for at least 3 months, followed by aspirin for life.

### Study End Points

All patients were followed up by clinical visit or telephone call. The primary end point was the incidence of clinically driven target lesion failure (TLF), a composite of cardiac death, target vessel myocardial infarction, and clinically driven target lesion revascularization (TLR). TLR was defined as any clinically driven repeat revascularization caused by a > 50% stenosis within DCB-treated segment and 5 mm proximal or distal to it. Target vessel thrombosis and bleeding were defined according to the Academic Research Consortium. All events were adjudicated by an independent clinical event committee.

### Statistical Analysis

Continuous variables are shown as mean ± SD or median (interquartile range), and dichotomous variables are expressed as counts and percentages of the total. Continuous variables were compared utilizing the unpaired Student’s *t*-test while categorical variables were compared using the Fisher’s exact test. All *p*-values were two-tailed, and values <0.05 were considered statistically significant. Cumulative event curves were calculated using the Kaplan–Meier method and compared using the log-rank test. Hazard ratios with 95% confidence intervals (CIs) were calculated using the Cox proportional hazard model. Factors with a *p*-value <0.2 on univariate analysis were entered into the multivariate Cox regression analysis. The mixed-effect model had been done, and the lesions from same patient were treated as random effect. All analyses were performed with SPSS version 24.0 (IBM, Munich, Germany).

## Results

### Baseline Characteristics of Patients

The study enrolled 119 patients with 138 *de novo* coronary lesions in vessels ≥2.75 mm. Of these, 66 patients (75 lesions) had bifurcation and 53 patients (63 lesions) had non-bifurcation lesions. The average age was 55.3 ± 10.8 years, and 72% patients were men. There were no significant differences in baseline demographic and clinical characteristics of patients in the bifurcation and non-bifurcation groups (Table 1).

**TABLE 1:** Baseline patient characteristics.

	All (*n* = 119)	Bifurcation (*n* = 66)	Non-bifurcation (*n* = 53)	*P-value*
Age (years)	55.3 ± 10.8	56.5 ± 10.1	53.7 ± 11.5	0.17
Male	86 (72%)	45 (68%)	41 (77%)	0.31
Current smoker	39 (33%)	23 (35%)	16 (30%)	0.69
Family history of CAD	5 (4%)	2 (3%)	3 (6%)	0.65
Diabetes mellitus	29 (24%)	18 (27%)	11 (21%)	0.52
Hypertension	61 (51%)	36 (55%)	25 (47%)	0.46
Hypercholesterolemia	64 (54%)	34 (52%)	30 (57%)	0.71
Renal failure*	1 (0.8%)	0	1 (2%)	0.44
Previous MI	9 (8%)	5 (8%)	4 (8%)	>0.99
Previous PCI	14 (12%)	8 (12%)	6 (11%)	>0.99
Ejection fraction (%)	61 (58-65)	61 (57-64)	62 (59-65)	0.21
**Procedure indication**				
STEMI	16 (13%)	8 (12%)	8 (15%)	0.79
Non-STEMI	9 (8%)	3 (5%)	6 (11%)	0.18
Unstable angina	83 (70%)	49 (74%)	34 (64%)	0.31
Stable angina	9 (8%)	6 (9%)	3 (6%)	0.73
Silent ischemia	1 (0.8%)	0	1 (2%)	0.44
**Periprocedural medications**				
DAPT (P2Y12 + Aspirin)	118 (99%)	66 (100)	52 (98%)	0.44
Statins	115 (97%)	65 (98%)	50 (94%)	0.32
β-blockers	83 (70%)	46 (70%)	37 (70%)	>0.99
ACE-I	59 (50%)	32 (48%)	27 (51%)	0.85

CAD, coronary artery disease; DAPT, dual antiplatelet treatment; LV, left ventricle; NSTEMI, non-ST elevation myocardial infarction; PCI, percutaneous coronary intervention; CABG, coronary artery bypass graft; STEMI, ST elevation myocardial infarction. *Renal failure was defined as an estimated glomerular filtration rate of <30 ml/min/1.73 m^2^. Values are mean ± SD, median (interquartile range), or % (n).

### Baseline Target Lesion and Procedural Characteristics

Target lesions were more common in left anterior descending (LAD) artery in the bifurcation group and right coronary artery (RCA) in the non-bifurcation group (Table 2). Bifurcation group had a large proportion of patients with American College of Cardiology/American Heart Association type B2 or C lesions (72.0 vs. 17.5%; *p* < 0.001). The percentage of true bifurcation lesions in the bifurcation group (Medina 1, 1, 1 or 1, 0, 1 or 0, 1, 1) was 24.0%. The average reference diameter was similar in the two groups (3.0 ± 0.3 vs. 3.1 ± 0.3; *p* = 0.27), but diameter stenosis was lower in the non-bifurcation group (82.6 ± 8.2% vs. 86.8 ± 7.9%; *p* = 0.003). To achieve adequate lesion preparation, cutting balloons were used in 85% lesions and there was no significant difference in the two groups (Table 3). The DCB length in the non-bifurcation group was slightly lower than that in the bifurcation group (*p* = 0.02), but the DCB diameter and DCB diameter/reference vessel diameter (RVD) ratio were similar in the two groups (*p* = 0.17 and 0.25, respectively). There were no significant differences between the two groups in DCB inflation time and deployment pressure. Three true bifurcation lesions were treated with DCB in both branches but no kissing balloon was used. Dissection occurred in 34 (24.6%) lesions after the lesion preparation. Most of those were type A or B, and were similar in both groups (*p* = 0.26). One patient in the bifurcation group experienced type C dissection, with no flow-limiting after DCB treatment and did not require bailout stenting. Residual stenosis was 30.3 ± 12.3% and 27.5 ± 11.1% in the bifurcation and non-bifurcation groups (*p* = 0.17).

**TABLE 2:** Baseline target lesion characteristics.

	All (*n* = 138)	Bifurcation (*n* = 75)	Non-bifurcation (*n* = 63)	*P-value*
Target Vessel				0.002
LAD/D	56.5%	65.3%	46.0%	
LCX/OM	28.3%	29.3%	27.0%	
RCA, PDA, PLV	15.2%	5.3%	27.0%	
**Bifurcation Medina Type**				
1,1,1 or 1,0,1or 0,1,1	13.0%	24.0%		
1,1,0 or 1,0,0 or 0,1,0 or 0,0,1	41.3%	76.0%		
ACC/AHA type B2/C lesion	47.1%	72.0%	17.5%	<0.001
Lesion Length* (mm)	14.2 ± 6.6	15.0 ± 7.2	13.2 ± 5.6	0.18
Reference vessel diameter* (mm)	3.1 ± 0.3	3.0 ± 0.3	3.1 ± 0.3	0.27
Diameter stenosis* (%)	85.0 ± 8.3	86.8 ± 7.9	82.6 ± 8.2	0.003

LAD/D, left anterior descending/diagonal branch; LCX/OM, left circumflex/obtuse marginal branch; RCA/PDA/PL, right coronary artery/posterior descending artery/posterior lateral; LMCA, left main coronary artery; CTO, chronic total occlusion; ACC/AHA, American College of Cardiology/American Heart Association. Values are mean ± SD, median (interquartile range), or % (n). *Visually estimated by operator.

**TABLE 3:** Procedural characteristics.

	All (*n* = 138)	Bifurcation (*n* = 75)	Non-bifurcation (*n* = 63)	*P-value*
Pre-dilation balloon diameter/RVD ratio	0.77 ± 0.09	0.77 ± 0.08	0.76 ± 0.10	0.62
Combining cutting balloon	84.8%	85.3%	84.1%	> 0.99
DCB diameter (mm)	2.9 ± 0.3	2.8 ± 0.3	2.9 ± 0.4	0.17
DCB diameter/RD ratio	0.93 ± 0.08	0.92 ± 0.08	0.94 ± 0.08	0.25
DCB length (mm)	21.0 ± 8.0	19.1 ± 9.5	23.5 ± 4.5	0.02
DCB inflation time (s)	54.2 ± 8.8	53.0 ± 9.9	55.8 ± 6.8	0.15
DCB deployment pressure (atm)	7.5 ± 1.6	7.5 ± 1.9	7.4 ± 1.3	0.45
Coronary dissection after DCB intervention (%)				0.26
Type A (%)	16.7%	16.0%	17.5%	
Type B (%)	7.2%	10.7%	3.2%	
Type C (%)	0.7%	1.3%	0	
Type D-F (%)	0	0	0	
Residual stenosis* (%)	29.1 ± 11.8	30.3 ± 12.3	27.5 ± 11.1	0.17

DCB, drug-coated balloon; RVD, reference vessel diameter. Values are mean ± SD, median (interquartile range), or % (n). *Visually estimated by operator.

### Clinical Outcomes

**FIGURE 1:**
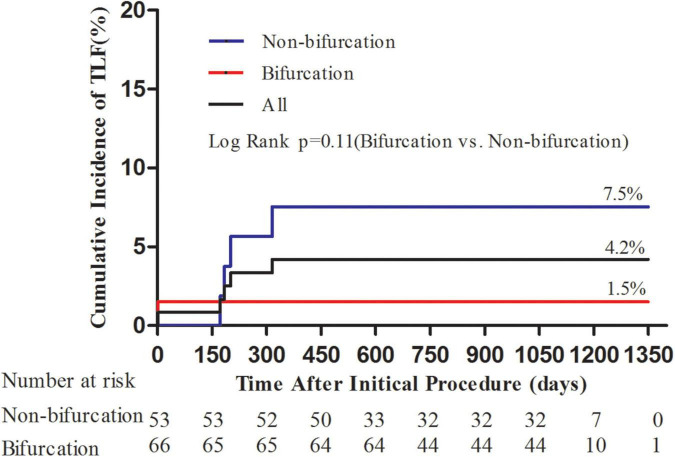
Kaplan–Meier curves for cumulative incidence of target lesion failure (TLF). Kaplan–Meier curves showed numerically lower TLF in the bifurcation group; however, it was not statistically different (*p* = 0.11).

**FIGURE 2:**
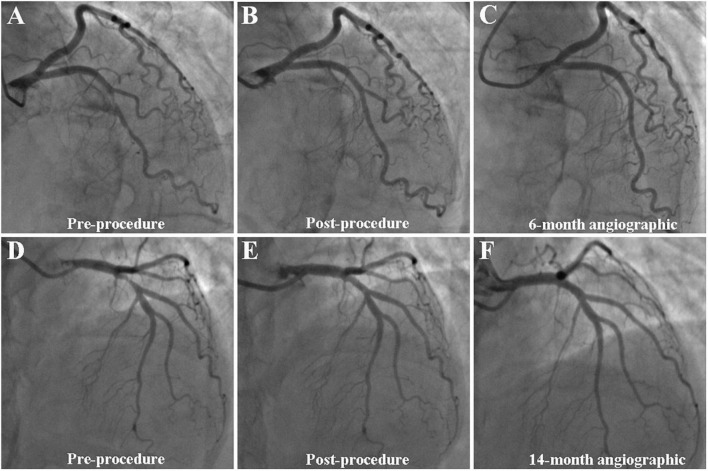
Case example of DCB-only strategy for bifurcation **(A–C)** and non-bifurcation **(D–F)**. **(A–C)** A case example of DCB-only strategy for bifurcation. **(A)** Initial angiogram with LAD 90% visual stenosis; **(B)** final result with 10% residual stenosis after DCB treatment; and **(C)** 6-month follow-up angiography showed no obvious stenosis. **(D–F)** A case example of DCB-only strategy for non-bifurcation. **(D)** Initial angiogram with LAD 90% visual stenosis; **(E)** final result with 20% residual stenosis after DCB treatment; and **(F)** 14-month follow-up angiography showed no obvious stenosis.

The average follow-up duration was 1.8 years with a median of 2 years. One patient lost follow-up at 1 year and two patients at 2 years. During hospitalization, one patient experienced periprocedural myocardial infarction. TLF occurred in five (4.2%), TLR in four (3.4%), and TVR in five (4.2%) patients (Table 4). Three out of four patients with TLR were treated again with DCB-only strategy and one with a DES. Although the frequency of TLR and TVR was higher in the non-bifurcation group (*p* = 0.04 and 0.02, respectively), there was no difference in TLF between the two groups (*p* = 0.17). As shown in Figure 1, the cumulative incidence of TLF (Kaplan–Meier estimates) in the two groups was not statistically different (log-rank [Mantel–Cox] *p* = 0.11). Table 5 provides the results of multivariate Cox regression analyses. The hazard ratio for TLF in the non-bifurcation vs. bifurcation group was 7.3 (95% CI: 0.4–144.3, *p* = 0.19) when adjusted for other covariates, including target vessel, diameter stenosis, DCB length, DCB diameter, lesion types, lesion length, DCB inflation time, and residual stenosis in the multivariate analysis. Case examples of DCB-only strategy for bifurcation and non-bifurcation are shown in Figure 2.

**TABLE 4:** Clinical outcomes.

Event	All (*n* = 119)	Bifurcation (*n* = 66)	Non-bifurcation (*n* = 53)	p* Value
TLF	5 (4.2%)	1 (1.5%)	4 (7.5%)	0.17
All cause death	0 (0%)	0 (0%)	0 (0%)	> 0.99
Cardiac death	0 (0%)	0 (0%)	0% (0)	> 0.99
MI	1 (0.8%)	1 (1.5%)	0 (0%)	>0.99
TLR	4 (3.4%)	0 (0%)	4 (7.5%)	0.04
TVR	5 (4.2%)	0 (0%)	5 (9.4%)	0.02
Non-TLR	2 (1.7%)	0 (0%)	2 (3.8%)	0.20
Non-TVR	1 (0.8%)	0 (0%)	1 (1.9%)	0.45
TV-thrombosis	0 (0%)	0 (0%)	0 (0%)	> 0.99

MI, myocardial infarction; TLF, target lesion failure; TLR, target lesion revascularization; TVR, target vessel revascularization; MACE, major adverse cardiac events; non-TLR, non-target lesion revascularization; non-TVR, non-target vessel revascularization; TV, target vessel. Values are % (n). *Bifurcation vs. non-bifurcation.

**TABLE 5:** Cox regression analysis of target lesion failure (TLF).

	Multivariate	
	
	Hazard ratio (95% CI)	*P-value*
Bifurcation	7.3 (0.4–144.3)	0.19
LCX/OM	0.9 (0.1–13.9)	0.91
RCA, PDA, PLV	1.9 (0.1–43.5)	0.67
Diameter stenosis	0.9 (0.9–1.1)	0.44
DCB length	1.1 (0.9–1.4)	0.43
DCB diameter	15.1 (0.5–411.3)	0.11
DCB inflation time	0.9 (0.8–1.0)	0.07
lesion type	0.6 (0.1–11.6)	0.72
lesion length	1.0 (0.8–1.2)	0.75
residual stenosis	1.0 (0.9–1.2)	0.70

*LCX/OM, left circumflex/obtuse marginal branch; RCA/PDA/PL, right coronary artery/posterior descending artery/posterior lateral; DCB, drug-coated balloon; CI, confidence intervals.*

## Discussion

To the best of our knowledge, this is the first specific study to evaluate the long-term efficacy of DCB-only approach for treating *de novo* lesions of large coronary vessels and compare bifurcation with non-bifurcation lesions. Our study demonstrated that (1) the TLF and TVR were low in *de novo* coronary lesions of large coronary vessels at long-term follow-up and (2) DCB-only strategy was safe and effective to treat *de novo* coronary bifurcation lesions of large coronary vessels.

Drug-coated balloons are semi-compliant balloons coated with antiproliferative agents that are released into the vessel wall after inflation (10). Once a coronary lesion has been adequately prepared without any significant residual stenosis and major dissection, then all what is needed is an antiproliferative agent to reduce neointimal proliferation and avoid post-angioplasty restenosis (11). Optimal lesion preparation is the key to successful DCB angioplasty. The recent consensus recommended a semi- or non-compliant with a balloon-to-vessel ratio of 1:1 (4). However, in our study, the pre-dilation balloon diameter/RVD ratio was about 0.77. More than 50% target vessels had severe stenosis and complex lesions, which may account for this phenomenon. Despite the use of cutting balloon in 85% lesions, no patient suffered major dissection or acute vessel closure requiring bailout stenting. This may be due to conservative balloon sizing and hence residual stenosis in 29%. Nevertheless, TLR rate remained low. Similar results have also been reported in recent randomized studies (12).

Evidence is growing for efficacy of DCB-only strategy in the treatment of *de novo* lesions in large coronary arteries. However, the follow-up time in most of these studies is less than 1 year. In our study, the average reference diameter was 3.1 ± 0.3 mm and the median follow-up time was 2 years. The incidence of TLF and TVR was low (both were 4.2%). Similar results have been shown in recent short- or mid-term studies. The DEBATE study enrolled 119 patients (135 *de novo* lesions) with reference vessel diameter 3.1 ± 0.3 mm. TLR and TVR at 12 months follow-up were 3.4 and 5.1%, respectively, with no cardiac death or MI (13). Similarly, another prospective study reported TLR and major adverse cardiac event (MACE) rates of 4.3%, with no MI or death (14). Interestingly, a retrospective study analyzed 222 patients with RVD ≥2.8 mm, and there was no TLR and MACE at an average of 10 months of follow-up (7). DCB-only strategy for native lesions of large coronary vessels has also shown promising results for specific indications, for example, patients with high bleeding risk or patients with complex acute coronary syndrome (15, 16). Moreover, the incidence of TLF with DCB was comparable to TLF with newer-generation DES. The TLF rates were 3.8 and 6.6% for the DCB and DES groups (*p* = 0.53) in PEPCAD NSTEMI trial (17). Similar results were also seen in the REVELATION study (18). However, this may be due to younger age of patients and relatively uncomplicated lesions treated in DCB studies. It is also postulated that the benefit of DCB may be time-dependent and studies with very long-term follow-up are needed to confirm it.

Coronary bifurcation lesions are still a challenge for PCI, with relatively lower success rate and higher complication rate (19). DCB-only strategy has a theoretical advantage of respecting the original anatomy of bifurcation, especially carina. It also avoids jailing the side branch. However, data for DCB-only in the treatment of coronary bifurcation lesions in large coronary vessels are limited. In an observational study, 39 patients with *de novo* bifurcation lesions and SB ≥2 mm were treated with DCB-only strategy (20). At 4-month follow-up, three patients (7.7%) suffered TLR and MACE, but no patient had cardiac death, MI, or stroke. Another observational study enrolled 70 patients (70 lesions), of whom 51 (73%) had true bifurcation lesions (21). The rates of TLR (4.5%) and TVR (6.1%) were low at 9-month follow-up. The randomized PEPCAD BIF trial compared DCB angioplasty to POBA in bifurcation lesion of Medina type 0, 0, 1 or 0, 1, 0 or 0, 1, 1 (22). Only one patient (3.1%) had TLR at 9-month follow-up. Although promising results have been shown in the above studies, most of them were either in small vessels or at short-term follow-up. Therefore, we explored the long-term efficacy of DCB-only strategy in bifurcation lesions of large vessels. Our study had 66 patients (75 lesions) with RVD 3.0 ± 0.3 mm, 24.0% with true bifurcation lesions, 72.0% with B2/C complex lesions, and 2-year follow-up. One patient experienced periprocedural myocardial infarction. Reassuringly, no patient suffered TLR and TVR in the bifurcation group. However, the cumulative incidence and Cox regression analysis of TLF were not statistically different between the two groups, which may represent relatively modest sample size. Although optimal approach for bifurcation PCI remains debatable (23, 24), DCB-only strategy offers an attractive and potentially effective alternative. A prospective randomized multicenter trial with a large number of participants is warranted to confirm the value of DCB-only in *de novo* bifurcation lesions of large coronary arteries.

## Study Limitation

There are several limitations. First, there was no routine angiographic follow-up in this study; however, one may argue that clinical outcomes are more important and relevant and indeed incidence of angina pectoris and TLR were low. Second, although the angiographic images for residual restenosis were reviewed by experienced interventionalists, the absence of quantitative coronary angiography assessment, which may lead to underestimation of residual stenosis. Third, as an observational study, selection biases cannot be excluded. Finally, although we studied outcomes at a median follow-up of 2 years, due to modest sample size, low incidence rates, and potential DCB benefits at very late time points, it remains desirable to study outcomes at even longer follow-up.

## Conclusion

This prospective observational study suggests that DCB-only strategy appears safe and effective for the treatment of both bifurcation and non-bifurcation lesions in native large coronary arteries.

## Data Availability Statement

The raw data supporting the conclusions of this article will be made available by the authors, without undue reservation.

## Ethics Statement

The studies involving human participants were reviewed and approved by the Ethical Review Board of Xuzhou Third People’s Hospital. The patients/participants provided their written informed consent to participate in this study.

## Author Contributions

Y-JZ and X-XT: supervisors of the study and guarantee the study data and accuracy. F-WH, SC, and QL: conception, design, analysis, and interpretation of data. F-WH, Y-JZ, and JI: drafting and revising of manuscript and final approval of the manuscript submitted. F-WH, Y-XZ, X-YW, Y-WC, Q-HZ, and BL: data collection, data analysis, and interpretation. All authors contributed to the article and approved the submitted version.

## Conflict of Interest

The authors declare that the research was conducted in the absence of any commercial or financial relationships that could be construed as a potential conflict of interest.

## Publisher’s Note

All claims expressed in this article are solely those of the authors and do not necessarily represent those of their affiliated organizations, or those of the publisher, the editors and the reviewers. Any product that may be evaluated in this article, or claim that may be made by its manufacturer, is not guaranteed or endorsed by the publisher.
